# Chloramine Disinfection-Induced Nitrification Activities and Their Potential Public Health Risk Indications within Deposits of a Drinking Water Supply System

**DOI:** 10.3390/ijerph17030772

**Published:** 2020-01-26

**Authors:** Xun Liu, Hong Liu, Ning Ding

**Affiliations:** 1 School of Civil Engineering, Suzhou University of Science and Technology, Suzhou 215000, China; liuxun8127@usts.edu.cn; 2 School of Environmental Science and Engineering, Jiangsu Key Laboratory of Environmental Science and Technology, Suzhou University of Science and Technology, Suzhou 215009, China; 3 Key Laboratory of Cleaner Production and Comprehensive Utilization of Resources, China National Light Industry, Department of Environmental Science and Engineering, Beijing Technology and Business University, Beijing 100037, China; dingning@btbu.edu.cn

**Keywords:** microsensors, deposit sediment, water supply, nitrification activity, diffusion

## Abstract

Microsensors were applied to study the diffusion reaction and activity of a nitrogen species of deposit sediment from a drinking water supply system. Microprofiles of dissolved oxygen (DO), NH_4_^+^-N, NO_3_^−^-N, and NO_2_^−^N in the sediment indicated that the DO concentration decreased from the highest at the sediment surface to zero at the bottom of the sediment. Similarly, with the increase of depth, NH_4_^+^-N initially increased rapidly and then decreased slowly, while the concentration of NO_3_^−^-N reached a maximum at around 6000 μm and then decreased to about 0.1 mg·L^−1^ near the bottom of the sediment. Almost no change was observed for NO_2_^−^-N. The decrease of NH_4_^+^-N and DO corresponded well with the increase of NO_3_^−^-N. Furthermore, based on a consumption and production rate analysis, DO has always been consumed; the NH_4_^+^-N consumption rate increased rapidly within 0–1000 μm, reaching about 14 mg·L^−1^·S^−1^·10^−9^. A small amount of NH_4_^+^-N was produced in 2000–6000 μm, which could be attributed to denitrification activity. There was no change deeper than 6000 μm, while NO_3_^−^-N was produced at a depth between 0 and 6000 μm and was consumed in the deeper zone. At the depth of 9000 μm, the NO_3_^−^-N consumption reached a maximum of 5 mg·L^−1^·S^−1^·10^−9^. The consumption of DO and NH_4_^+^-N, which corresponded with the production of NO_3_^−^-N in a specific microscale range within the sediment, demonstrated nitrification and denitrification activities. In addition, the time required for the diffusion of only DO, NH_4_^+^-N, NO_3_^−^-N, and NO_2_^−^-N was estimated as 14 days; however, in the practical, even after 60 days of operation, there was still a continuous reaction, which provided further evidence towards microbial activities within the sediment.

## 1. Introduction

Various water quality problems in water supply networks remain a huge challenge for water supply industries around the world. Due to the concerns with disinfection by-products (DBPs) and stringent limits on DBPs in drinking water systems, more and more water plants are using chloramine as a secondary disinfectant instead of chlorine disinfection. For example, many water treatment plants in the United States have gradually shifted from chlorine disinfection to chloramine disinfection in order to meet the requirements of the disinfection/DBPs regulations. Several European countries also use chloramine as a final disinfectant. This aspect has led to different public health issues, with a request for derogations from the water quality standards [1,2]. However, water supply systems using chloramine disinfection generally have water quality problems that are caused by biological nitrification [3,4]. Firstly, due to the decay of chloramine, ammonia nitrogen will be released into the water, and incomplete nitrification will lead to the accumulation of nitrite nitrogen, which brings about potential human health hazards [5,6]. Secondly, biological nitrification can promote the growth of ammonia-oxidizing bacteria (AOB) and nitrite-oxidizing bacteria (NOB) and promote the formation of pipe network biofilm or sediment, providing a more proper environment for the large-scale reproduction of bacteria and thereby reducing the biological stability of drinking water [7,8]. In addition, nitrification will consume a large amount of dissolved oxygen, lower the pH value, and accelerate pipeline corrosion, resulting in "red water" problems [9].

Although the control of nitrification in drinking water piping networks has attracted widespread public attention, most current research focuses on the macroscopic control of nitrification in pipe networks, including the formation of pipe network biofilm and the factors that influence nitrification. Water treatment plants typically control the growth of bacteria in the pipe network by adding chlorine and maintaining a certain amount of residual chlorine at the ends of the pipe network. However, maintaining the amount of residual chlorine in the water distribution network does not necessarily control the growth and reproduction of bacteria in the biofilm or sediment of the pipe network. LeChevallier [10] found that even with sufficient residual chlorine (3 mg·L^−1^), the growth and change in activity of biofilm in a pipe network system cannot be effectively controlled. On the one hand, the rate of chloramine decay in the presence of biofilm is about half of that in tap water [11], and the presence of the biofilm leads to a decrease in the disinfectant molecules that can diffuse into the interior of the biofilm. On the other hand, nitrifying bacteria are widely propagated in the distribution network of drinking water disinfected by chloramine [12,13,14]. The formation of biofilms or sediments in the pipeline and a large number of nutrients in the pipe network that can be used by nitrifying bacteria are beneficial to the survival of nitrifying bacteria in a water supply system disinfected with chloramine. Nitrifying bacteria in the attached state are much more (2 to 100 times) resistant to disinfectants than nitrifying bacteria in the suspended state [15]. These precipitations or sediments provide a habitat for the growth and reproduction of nitrifying bacteria, and the nitrifying bacteria are protected by sediments to avoid the inactivation of disinfectants [16,17].

In order to control nitrification and decrease the interaction of AOB in the water and AOB in the biofilm of the pipe wall [18], researchers have investigated factors that affect nitrification activities, including pH, water temperature, chloramine concentration, ammonia nitrogen concentration, organic matter in the water, the hydraulic retention time of the pipe network, the pipeline’s properties, biofilm of the pipe wall, and the disinfection process [19,20,21,22,23,24]. Other researchers have studied the diversity of nitrifying bacteria in the network from the perspective of microbial characteristics, and have also studied the relationship between different bacteria and disinfectant concentration [25]. Studies [26] have shown that the presence of AOB is almost undetectable in water that is treated with chloramine in water plants. However, using molecular biology techniques to analyze the community structure of nitrifying bacteria, it was found that the dominant community in the ammonia-oxidizing bacteria population (*Nm. Oligotropha*) exists at the end of the pipe network. Some other studies have used microelectrodes to analyze the distribution of chemical parameters in the biofilm of drinking water networks. De Beer has developed chlorine microelectrodes and used them to measure the chlorine permeability of biofilms [27]; Lee and Pressman et al. prepared a chlorine microelectrode that can be used to measure chloramines in biofilms, and studied the penetration of free chlorine and chloramines into biofilms by free chlorine and chloramine microelectrodes [28,29,30,31].

Based on previous research, it can be seen that these relationships between nitrifying bacteria and disinfectants hidden in the biofilm or sediment of the pipe network are not clear. Little research has been conducted into the biological nitrification activities or the diffusion of nitrogen species in the microscopic environment of the pipe network. Thus, studies on the diffusion reaction and nitrification biological activities in the microenvironment of the pipe network have high theoretical value and practical significance for ensuring water supply safety.

In this study, we analyzed the diffusion of nitrogen species and nitrification activities in the microscopic environment of sediments in water supply networks. Microsensors with tip diameters as small as several micrometers were used to obtain the concentration profiles of characteristic parameters, including ammonia nitrogen, nitrate, nitrite, and dissolved oxygen, in the vertical direction of the sediment’s microenvironment. The concentration distribution of the nitrogen species in the sediment’s microenvironment and its relationship with biological nitrification activities may be linked.

## 2. Materials and Methods

### 2.1. Sample Preparation

A deposit sediment sample (provided by Wuxi Zhongqiao Drinking Water Treatment Plant, Jiangsu Province, China) with a size of 2 cm (20,000 µm) was placed in a sterilized glass cup with dimensions of 6 cm (diameter) × 6 cm (depth). Disinfectant monochloramine (4 mg·L^−1^) was continuously flowing into the reactor, and the flow rate was kept at 4 mL·min^−1^. The reactor was operated at room temperature (21–23 °C) under steady-state conditions (pH 8.0, 5 mM boric acid/sodium hydroxide buffer solution, a 4 mL·min^−1^ flowrate, and 4 mgCl_2_·L^−1^ monochloramine). Microsensor profiles of dissolved oxygen (DO), NH_4_^+^-N, NO_3_^−^-N, and NO_2_^−^-N were measured.

### 2.2. Microsensor Fabrication

A combined amperometric O_2_ microsensor was developed based on previous studies [32,33,34]. Calibration of the O_2_ microsensor was performed with N_2_ and pure O_2_. Information on the fabrication and calibration of the NH_4_^+^-N, NO_3_^−^N, and NO_2_^−^-N microsensors can be found in [35,36].

### 2.3. Microsensor Measurements

Each microsensor was calibrated before and after measurements. During measurements, microsensors were mounted on a micro-manipulator (Model M3301R, World Precision Instruments, Inc., Sarasota FL, USA). Firstly, the microsensor’s tip was placed above the water cap of the reactor. Through controlling the micro-manipulator, the microsensor was moved towards the sediment surface, which was observed through the microscope (Model: Stemi SV11, Carl Zeiss, Jena, Germany). The step size of 100 to 200 μm was selected with enough resolution for the 2 cm sediment measurement.

### 2.4. Flux Calculation

Production and consumption rates of DO, NH_4_^+^-N, NO_3_^−^-N, and NO_2_^−^-N were calculated based on Fick’s second law of diffusion [37,38,39], which is shown in the following equation:

(1) $$\frac{\partial C_{\left(z,t\right)}}{\partial t}=D_{s}\times \frac{\partial^{2}C_{\left(z,t\right)}}{\partial z^{2}}-R_{\left(z\right)}+P_{\left(z\right)}$$

where *C*(*d*,*t*) stands for the concentration at time *t* and depth *d*, Ds represents the diffusion coefficient, *R* is the consumption rate, and *P* is the production rate.

Assuming that the reaction was at a steady state:

(2) $$\frac{\partial C_{\left(d,t\right)}}{\partial t}=0$$

Equation (l) can be rewritten as:

(3) $$Activity\left(d\right)=D_{z}\times \frac{\partial^{2}C_{\left(d,t\right)}}{\partial z^{2}}=R\left(d\right)-P\left(d\right)$$

where A(*d*) is the activity at depth z. A negative A(*d*) value reflects net production activity and a positive A(*d*) reflects net consumption activity. The concentration profiles were analyzed mathematically by means of a discrete version of Fick’s first law:

(4) $$J_{\left(d+1/2\Delta d\right)}=D_{d}\frac{C_{\left(d+\Delta d\right)}-C_{\left(d\right)}}{\Delta d}$$

where J(*d*+1/2∆*d*) is the flux at the depth between two data points, C is the concentration, and ∆d is the vertical distance between the two data points.

The D (diffusion coefficient) of NH_4_^+^, NO_3_^−^, and DO was 1.38−10^−5^ cm^2^∙s^−1^, 1.23 × 10^−5^ cm^2^∙s^−1^, and 2.09 × 10^−5^ cm^2^·s^−1^, respectively [40]. A flux profile was derived from the concentration profile using Equation (4). The activity profile was then derived from the flux profile:

(5) $$A_{\left(d\right)}=\frac{\left[J_{\left(d-1/2\Delta d\right)}-J_{\left(d+1/2\Delta d\right)}\right]}{\Delta d}$$

### 2.5. Diffusion Analysis

In order to investigate the chemical diffusion rate within the sediment, a simple case with the assumption that no reactions were occurring and a nonlinear equation [41] was used to simulate the chemical diffusion time within the sediment. The determination of the expected time of chemical diffusion was calculated based on the following:

(6) $$\frac{C-C_{0}}{C_{1}-C_{0}}=1-\frac{4}{\pi }\sum_{n=0}^{\infty }\frac{{\left(-1\right)}^{n}}{2n+1}exp\left\{\frac{-D{\left(2n+1\right)}^{2}\pi^{2}t}{4l^{2}}\right\}cos\left(\frac{\left(2n+1\right)\pi x}{2l}\right)$$

In Equation (6), l is the sediment depth and diffusion length. d represents the distance above an impermeable base; for example, d is defined as 0 μm at the bottom of the sediment, and d equals 20,000 μm at the sediment surface. C represents the concentration at location d, C_0_ is the concentration in the liquid layer, C_1_ is the constant concentration in the bulk water and is equivalent to C_s_, D is the diffusion coefficient, and t represents time.

In the case that only diffusion occurs within the sediment—for example, if chemicals are diffusing from the liquid layer into the sediment—C_0_ should be zero, so Equation (6) can be written as $\frac{C-C_{0}}{C_{1}-C_{0}}=\frac{C}{C_{1}}=\frac{C}{C_{s}}$. If chemicals are diffusing from the sediment into the liquid layer, C_0_ is a known value and the surface concentration is assumed to be zero, C_1_ = C_s_ = 0, and thus Equation (6) can be rewritten to Equation (7), which was implemented in R software to obtain the estimated diffusion time.

(7) $$\frac{C-C_{0}}{C_{1}-C_{0}}=\frac{C-C_{0}}{-C_{0}}=-\frac{C}{C_{0}}+1$$

## 3. Results and Discussion

### 3.1. Microsensor Measurements of DO and Nitrogen Species

**Figure 1:**
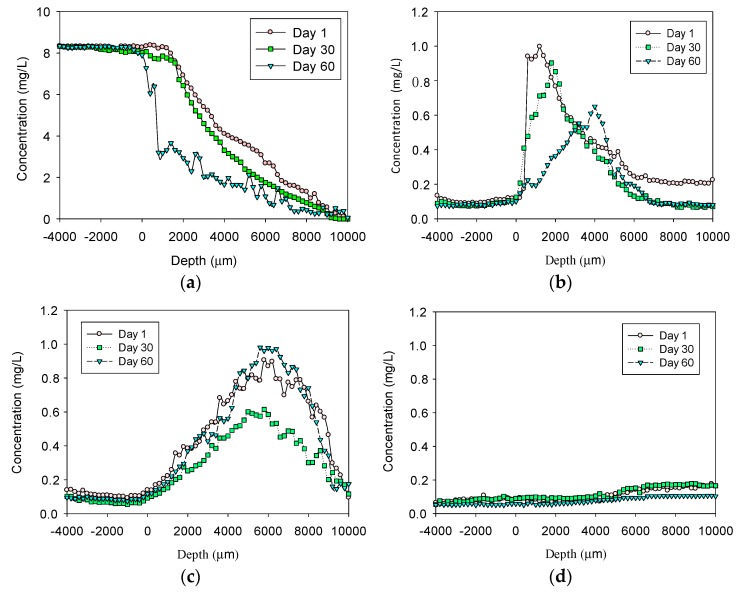
Microsensor profiles within the 10,000 μm sediment layer: (**a**) dissolved oxygen (DO); (**b**) NH_4_^+^-N; (**c**) NO_3_^−^-N; and (**d**) NO_2_^−^-N.

Microsensor profiles of DO, NH_4_^+^-N, NO_3_^−^-N, and NO_2_^−^-N in the sediment are shown in Figure 1. It can be seen in Figure 1a) that the DO concentration decreased from the highest value near the sediment surface to zero near the bottom of the sediment. For example, at Day 1, at 6000 μm below the interface between the water and the sediment, the DO value dropped from 8.3 mg·L^−1^ to about 2.68 mg·L^−1^; at Day 30, at the same depth of 6000 μm, the DO dropped to 1.75 mg·L^−1^; at Day 60, at the same depth of 6000 μm, the DO became 1 mg·L^−1^. At Day 60, within 1000 μm, the DO dropped sharply to about 3.18 mg·L^−1^ and continuously decreased to 1.09 mg·L^−1^ at 6000 μm, indicating a rapid consumption of DO and potential oxidation activity. Meanwhile, the concentration of NH_4_^+^-N (Figure 1b) initially increased rapidly and then decreased slowly. The microsensor profile’s tendency for Day 1 and Day 30 were similar; the NH_4_^+^-N concentration reached a maximum of approximately 1.0 mg·L^−1^ at around 2000 μm below the interface and then decreased to zero near the bottom of the sediment. At Day 60, the maximum value decreased from about 0.6 mg·L^−1^ to about 0.2 mg·L^−1^ around 6000 μm before slowly decreasing. Correspondingly, the concentration profiles of NO_3_^−^-N (Figure 1c) increased first and then decreased, indicating the production of nitrate due to nitrification. They then decreased to the deeper zone of the sediment, where DO was less than 2 mg·L^−1^, which could be attributed to denitrification activity. There was no significant change for NO_2_^−^-N (Figure 1d).

### 3.2. Estimation of Production and Consumption Rates

**Figure 2:**
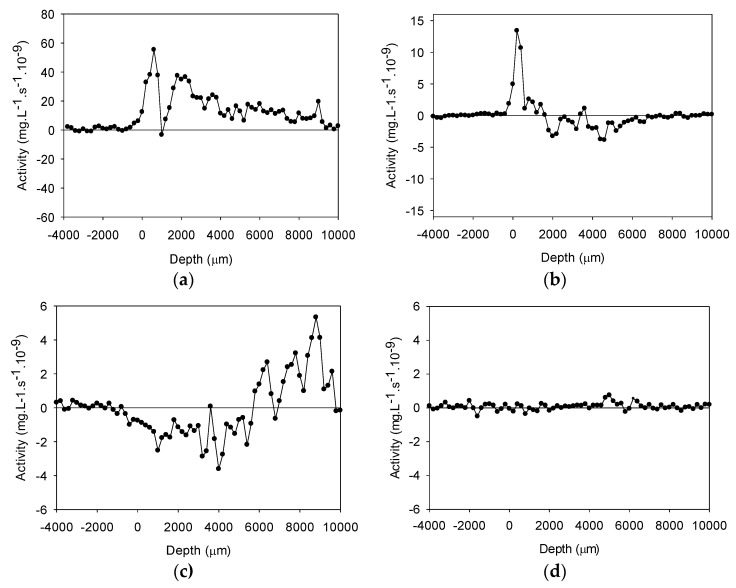
Activity profiles (a positive value represents consumption while a negative value represents production) of: (**a**) DO; (**b**) NH_4_^+^-N; (**c**) NO_3_^−^-N; and (**d**) NO_2_^−^-N.

Figure 2 shows the net specific consumption and production rates of DO, NH_4_^+^-N, NO_3_^−^-N, and NO_2_^−^-N. As seen in Figure 2a, DO was consumed across the whole sediment sample, and the consumption of DO decreased gradually with the increase of depth. NH_4_^+^-N consumption (Figure 2b) increased rapidly within 0–1000 μm, and reached about 14 mg·L^−1^·S^−1^·10^−9^ at 200 μm. A small amount of NH_4_^+^-N was produced at 2000–6000 μm, which may be attributed to denitrification, while NO_3_^−^-N was produced in the range of 0–6000 μm and consumed in the range of 6000–10,000 μm (Figure 2c). At the depth of 9000 μm, the consumption rate reached a maximum value of 5 mg·L^−1^·S^−1^·10^−9^. The consumption and production of NH_4_^+^-N and NO_3_^−^-N directly reflect that nitrification occurred in the oxic zone of the sediment, while denitrification was expected in the anoxic area in the deeper zone. Almost no change was observed for NO_2_^−^-N, as shown in Figure 2d, which indicates that full nitrification occurred within the sediment.

### 3.3. Diffusion Analysis

**Figure 3:**
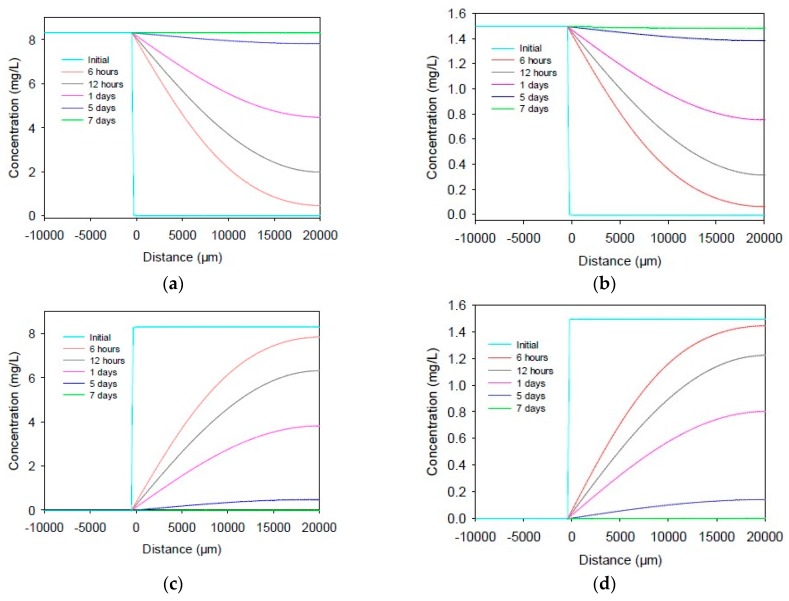
Simulated time required for diffusion into the sediment: (**a**) DO; and (**b**) NH_4_^+^-N and NO_3_^−^N. Simulated time required for diffusion out of the sediment: (**c**) DO; and (**d**) NH_4_^+^-N and NO_3_^−^-N.

When considering the diffusion and reaction of nitrogen species within the sediment, it is quite important to estimate the diffusion time through the sediment without reactions. In the case of diffusion only and using water diffusion coefficients for each chemical, the model estimated the times required for reaching the surface or bottom of the sediment, as shown in Figure 3. It is noted that almost no nitrite nitrogen was produced or detected by the microsensors; therefore, only ammonium nitrogen and nitrate nitrogen diffusion were simulated in the present study.

For example, the diffusion time required for 8.3 mg·L^−1^ of DO within the sediment and for 1.5 mg·N·L^−1^ of ammonium or nitrate both were estimated as seven days. A measurable concentration of DO, ammonium nitrogen, and nitrate nitrogen at the bottom of the sediment would be expected after approximately six hours. Complete diffusion of these chemicals out of the sediment would also be expected to be accomplished after approximately seven days (one week).

It is noted that diffusion occurred through the pores of sediment in the practical; therefore, the diffusion coefficient values in sediment (D_s_) were usually estimated as twice that in water. As a result, the required diffusion time would be twice that needed in the water phase. Therefore, a measurable concentration of DO, ammonium nitrogen, and nitrate nitrogen at the bottom of the sediment would be expected after approximately 12 hours. Full diffusion of these chemicals out of the sediment was expected to finish after approximately 14 days (two weeks). Compared to the microsensor profiles shown in Figure 1, it is obvious that not only did diffusion occur within the sediment, for example in Figure 1a, but even after six months, there remained around 6 mg·L^−1^ of DO at the interface of the sediment, and, as shown in Figure 1c, nitrate was always present but did not diffuse out of the sediment at either Day 30 or Day 60, demonstrating active biological reactions within the sediment.

## 4. Conclusions

Microelectrodes of DO, NH_4_^+^-N, NO_3_^−^-N, and NO_2_^−^-N were successfully used to obtain the gradient profiles within precipitated deposits from a drinking water supply system. The decrease and consumption of DO and NH_4_^+^-N accompanied by the increase and production of NO_3_^−^-N indicated nitrification activities within the sediment deposit. Nitrification tended to occur within the oxic zone of the sediment, while denitrification occurred in the deeper anoxic zone. The complete diffusion of DO and nitrogen species was not observed, which indicated that microbial functions were active. A measurable concentration of DO, NH_4_^+^-N, and NO_3_^−^-N at the bottom of the sediment would be expected after approximately 12 hours, and full diffusion would occur after approximately 14 days. The present study contributes to our understanding of nitrification activities within the microenvironment of sediment deposits, allowing for a better understanding of biochemical mechanisms in drinking water supply networks. The microbial activities remained active even after several months’ disinfection, which indicated the potential for public health risks and water safety issues within drinking water supply systems. Future studies on the release of chemicals or microorganisms from deposits into the water phase need to be performed. Further studies need to be conducted for the strategic control of biological stability. In practice, it is necessary to perform regular inspections and cleaning of the deposits from the distribution networks to avoid public health risks due to the potential release of microorganisms from deposits into the water phase.
